# Neuromuscular junction formation by compartmentalized culture using human iPS cell-derived motor neurons and skeletal muscle cells

**DOI:** 10.3389/fncir.2026.1838276

**Published:** 2026-09-17

**Authors:** Masayoshi Kamon, Moe Yamashita, SokeLee Siew, Shuji Wakatsuki, Toshiyuki Araki

**Affiliations:** 1 Department of Peripheral Nervous System Research, National Institute of Neuroscience, National Center of Neurology and Psychiatry, Tokyo, Japan; 2 Department of Biotechnology and Life Science, Graduate School of Engineering, Tokyo University of Agriculture and Technology, Tokyo, Japan

**Keywords:** culture modeling, imaging, induced pluripoten stem cell, motor neuron (MN), MyoD, neuromuscular junction (NMJ), synaptogeneisis, skeletal muscle cell (SMC)

## Abstract

The neuromuscular junction (NMJ) is a synapse that connects the nerve terminal of a motor neuron to a skeletal muscle fiber. The mechanisms underlying NMJ formation have been elucidated primarily through studies in animal models. However, elucidation of the pathophysiological mechanisms of human neuromuscular diseases and identification of potential therapeutic agents require the establishment of a human NMJ model. In this study, we established a human NMJ model using a compartmentalized culture system with cells derived from human induced pluripotent stem (iPS) cells, which enables quantitative and reproducible assessment of NMJ formation. During extended NMJ culture, morphological changes in the NMJ were observed, along with alterations in the expression of genes related to NMJ and skeletal muscle development, suggesting progressive NMJ maturation. The functionality of the formed NMJs was also confirmed using fluorescent live-cell imaging. These findings suggest that this human NMJ culture model may be applicable for investigating the pathological mechanisms of human neuromuscular diseases and exploring therapeutic agents.

## Introduction

NMJs are chemical synapses formed between the axons of motor neurons (MNs) and skeletal muscle cells (SMCs). The cell bodies of MNs are located in the midbrain and spinal cord within the central nervous system and extend their axons to the peripheral muscles. Mature NMJs exhibit a characteristic morphology in which the motor neuron axon terminal and the invaginated surface of the SMC membrane face each other across the synaptic cleft. Presynaptic terminals of MNs contain numerous synaptic vesicles filled with the neurotransmitter acetylcholine, which is released in response to neuronal excitation. Acetylcholine binds to acetylcholine receptors (AChRs) clustered on the postsynaptic membrane of SMCs, triggering postsynaptic signaling and generating an action potential in the muscle cell (Hall and Sanes, 1993; Sanes and Lichtman, 1999). This action potential leads to the opening of Ca^2+^ channels in the transverse tubules, resulting in the release of Ca^2+^ from the sarcoplasmic reticulum and ultimately inducing muscle contraction (Sanchez and Stefani, 1978; Somlyo et al., 1981).

NMJ is one of the most extensively studied synapses and has served as an important model for understanding the principles that regulate synaptogenesis, neural circuit formation during development, and the maturation and maintenance of synapses. Previous studies have shown that AChRs, together with postsynaptic-like structures, are spontaneously formed around the center of muscle fibers, a process known as muscle prepatterning (Yang et al., 2001; Arber et al., 2002; Panzer et al., 2006). This prepatterning process involves the assembly of the MuSK–LRP4–Dok-7–rapsyn complex, which promotes AChR clustering. Subsequently, agrin, a master organizer of NMJs, is produced and secreted from presynaptic terminals. The MuSK–LRP4–Dok-7–rapsyn complex functions as a receptor complex for agrin and induces the assembly and stabilization of postsynaptic NMJ structures, including AChR clusters, while promoting the dispersal of non-synaptic AChRs (Kummer et al., 2006; Kim et al., 2008). As NMJs mature, AChR clusters adopt increasingly branched morphologies, forming the characteristic “pretzel-like” structures (Sanes and Lichtman, 1999). In molecular levels, the fetal form of AChRs, containing a *γ* subunit (composition α2βγδ) is gradually replaced by an ε subunit-containing adult form (α2βδε) in the course of this maturation (Mishina et al., 1986). This morphological and molecular NMJ maturation is accompanied by differentiation of the myotube, including changes of myosin heavy chain subunits (Schiaffino and Reggiani, 2011). Abnormalities of the NMJ caused by mutations in genes encoding molecules involved in NMJ formation and signal transduction represent one of the most common pathological causes of congenital myasthenic syndrome (CMS) in humans, which is characterized by muscle weakness and a reduced safety margin for synaptic transmission (Zhang et al., 2023; Ohno et al., 2025). The regression of the NMJ over aging is a contributing factor to muscle atrophy and locomotive syndrome in old age (Gonzalez-Freire et al., 2014; Liu et al., 2017).

The mechanisms underlying NMJ formation have been studied using models from a variety of species, including rodents, chick, zebrafish, and Drosophila (Shimada et al., 1969; Peterson and Crain, 1970; Broadie and Bate, 1993; Nguyen et al., 1999). Among them, rodent models have been employed for NMJ studies in mammals. However, recent studies have reported that human NMJs are morphologically distinct from those in rodents (Jones et al., 2017). Human NMJs tend to exhibit a more fragmented or patchy morphology and show an increase in AChR cluster area with aging (Oda, 1984). These findings suggest that the processes governing synaptic maturation in humans may differ from those in rodents. To elucidate the mechanisms of NMJ formation and maturation in humans, as well as the pathogenesis of diseases involving NMJ dysfunction such as CMS, it is essential to develop experimental systems that model NMJ formation using human-derived cells.

In recent years, coculture systems using MNs and SMCs differentiated from human induced pluripotent stem cells (iPSCs) have been reported as platforms for modeling NMJ formation in humans (Demestre et al., 2015; Yoshida et al., 2015; Lin et al., 2019). Furthermore, human *in vitro* models using organ-on-chip technology, organoids, and assembleroids have also been reported (Andersen et al., 2020; Faustino Martins et al., 2020; Osaki et al., 2020; Yin et al., 2025). Although several studies have demonstrated the formation of NMJs using cocultures of human stem cell–derived MNs and SMCs, it usually takes a few months to observe the NMJ formation, and the performed analyses are mostly on the morphology and functionality of the formed NMJs. On the other hand, the quantitative reproducibility of NMJ formation—an essential requirement for evaluating therapeutic interventions in disease models—has not been sufficiently established.

Here, we established a novel experimental model of NMJ formation *in vitro* using a coculture system of MNs and SMCs differentiated from human iPSCs (Figure 1A). Our protocol enables quantitatively reproducible formation of morphologically identifiable NMJ structures in culture. During the course of coculture, enlargement of NMJs, together with increased expression of AChR subunits, indicative of NMJ maturation, was observed with extended culture periods. Furthermore, stimulation of MNs connected to SMCs induced an increase in intracellular Ca^2+^ levels in the SMCs, suggesting that the morphologically identifiable NMJ structures are functionally active. These findings indicate that our human cell–based NMJ formation system provides a useful platform for studying human NMJ development and its associated pathological conditions.

**Figure 1:**
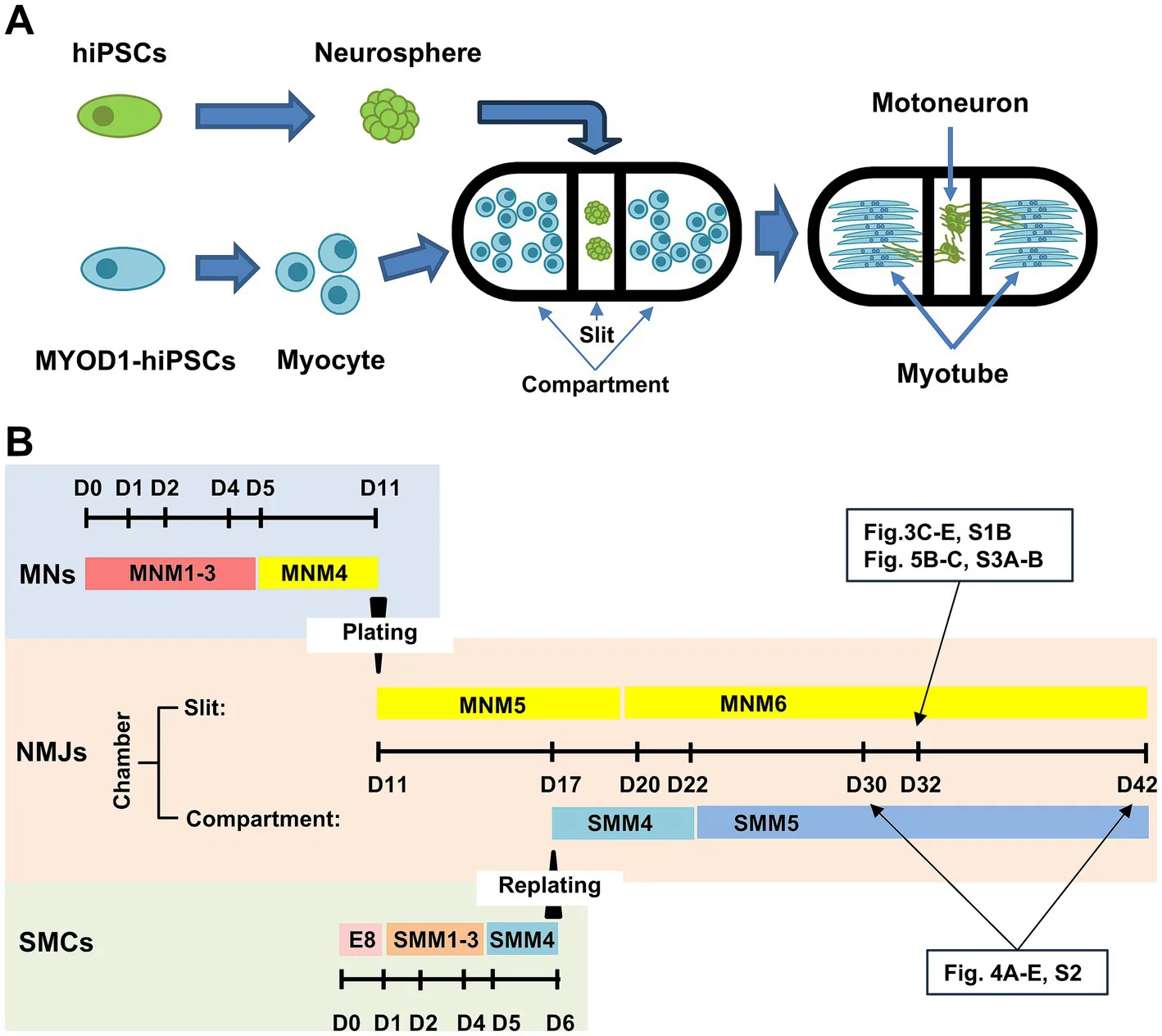
NMJ formation culture using human iPSC-derived motor neurons and myocytes. **(A)** Schematic diagram of NMJ formation culture using the compartmentalized culture system. **(B)** Schematic representation of the entire time course of NMJ formation culture. MNM, MN differentiation medium; SMM, SM differentiation medium (see Supplementary Tables S1–S4).

## Methods

### Antibodies

The following antibodies were used in this work. NFM (1:1000, AB1987, Millipore), HB9 (1:100, DSHB), ISL (1:100, DSHB), MHC (1:400, MAB4470, R&D Systems), SF (1:200, 101,002, Synaptic Systems), Goat anti-mouse IgG Alexa594 (1:500, A-11005, Thermo Scientific), Goat anti-mouse IgG Alexa488 (1:500, A-11001, Thermo Scientific), Goat anti-rabbit IgG Alexa488 (1:500, A-11008, Thermo Scientific), and Goat anti-mouse IgG Alexa405 (1:500, A-31556, Thermo Scientific).

### Human iPSC culture and differentiation

The use of human iPSCs was performed under approval of National Center of Neurology and Psychiatry Ethics Committee. The human iPS cell line 454E2 was provided by the Center for iPS Cell Research and Application, Kyoto University, Japan (Okita et al., 2011). Human iPSCs were cultured on vitronectin (A14700, Thermo Scientific)-coated dishes in Essential 8 Flex (E8) medium (A28585, Thermo Scientific) according to the manufacturer’s protocol. Cells were passaged every 3–5 days using 0.5 mM EDTA (15105–35, Nacalai Tesque) and plated in E8 medium containing 10 μM Y-27632 (Y-5301, LC Laboratories).

For MN differentiation, 454E2 iPSCs were cultured according to previously reported protocols with minor modifications (Maury et al., 2015; Shimojo et al., 2015). The iPSCs were first cultured in CDM (Kohjin Bio) containing 10 ng/mL bFGF (RCHEOT002, ReproCELL), 3% KSR (10828028, Thermo Scientific), and 20 ng/mL Activin A (338-AC, R&D Systems) to adapt them to MNM1. The adapted cells were dissociated using 0.01% trypsin/EDTA (T4049, Sigma) and counted. Cells were seeded into low-attachment V-bottom 96-well plates (81100675, Thermo Scientific) at 0.9–2.0 × 10^4^ cells per well in MNM1 to form neurospheres. Two days later, the culture medium was changed to MNM2 medium. Two days after the culture medium change, neurospheres were transferred to low-attachment 90-mm dishes (174945, Thermo Scientific) and cultured in MNM3. The following day, the medium was changed to MNM4. After 11 days of differentiation, the spheres were dissociated using Accumax (AM-105, Innovative Cell Technologies) and seeded onto Matrigel (356231, Corning)-coated 24-well plates at 1 × 10^5^ to 1 × 10^6^ cells per well. The next day, the cells were cultured in MNM5. After 20 days of differentiation, the medium was changed to MNM6. Cells were analyzed 21 days or more after the initiation of NMJ formation.

For skeletal muscle cell (SMC) differentiation, a doxycycline (Dox)-inducible MYOD1 expression vector was electroporated into 454E2 iPSCs using NEPA21 (Nepa Gene) (Uchimura et al., 2017). Transduced cells were selected with 1 μg/mL puromycin (P8833, Sigma), and clones exhibiting uniformly high MYOD1 expression were selected for SMC differentiation. SMC differentiation was performed as previously described, with minor modifications (Tanaka et al., 2013; Mendjan et al., 2014; Uchimura et al., 2017; Akiyama et al., 2018). The MYOD1-inducible iPSCs were dissociated using 0.01% trypsin/EDTA and counted. Cells were seeded onto Matrigel-coated 100-mm dishes at 0.5 × 10^6^ to 1 × 10^6^ cells per dish. The following day, the medium was changed to SMM1, followed by SMM2 2 days later. After 3 days of differentiation, the cells were cultured in SMM3. One day later, the medium was changed to SMM4. After 6 days, the cells were replated onto Matrigel-coated 24-well plates using Accutase (12679–54, Nacalai Tesque) at 0.5 × 10^6^ to 1 × 10^6^ cells per well and maintained in SMM4 for an additional 5 days.

### NMJ formation by compartmentalized culture

Matrigel-coated 60 mm dishes were scratched by Pin Rake (Tyler Research Corporation) for direction of neurite extension, and Campenot chamber (Tyler Research Corporation) with grease (MOLYKOTE) was attached over the scratch channels (Campenot, 1977). The grease is impermeable to the culture medium, preventing mixing between the media in the “slit” and the “chambers.” However, axons extending from the spheres in the “slit” can grow along the scratched paths, pass through the grease barrier, and reach the chamber compartments, where they contact SMCs.

The dispersed neurospheres after 11 days of MN differentiation were seeded into the slit of the chamber. All culture compartments were filled with MNM4. The medium was changed to MNM5 the next day and maintained for another 5 days. SMCs (3 × 10^5^ to 5 × 10^5^ cells) from day 6 of the SMC differentiation protocol were seeded in the chamber compartment. The next day, the medium in “compartments” was switched to SMM4, and the medium outside of the “compartments” (i.e., MN medium) was changed to MNM5. On the 9th day of the compartmentalized culture, the medium in the “compartments” was changed to SMM4, and the medium outside the “compartments” was changed to MNM6. On the 11th day of the compartmentized culture, the medium in the “compartments” was changed to SMM5, and the medium outside the “compartments” was changed to MNM6. The cultures were maintained until examinations on days 30, 32, and 42.

### Immunocytochemistry

Cells were fixed with 4% paraformaldehyde (PFA) (26126–25, Nacalai tesque) for 10 min at room temperature. After fixation, the cells were permeabilized with 0.3% Triton X-100 for 5 min at room temperature. After blocking with 3% FBS for 1 h, the cells were incubated overnight at 4 °C with a primary antibody, then for 1 h at room temperature with a fluorescent dye-conjugated secondary antibody. For visualization of NMJ, cells were incubated with 1 μg/mL tetramethylrhodamine (TMR) conjugate *α*-BTX (T1175, Thermo Scientific) in a CO_2_ incubator for 30 min (Yumoto et al., 2008, 2012). Subsequently, the cells were fixed with 1% PFA for 30 min at room temperature and subjected to the immunocytochemical procedures. Cell nuclei were counterstained with DAPI and observed under a fluorescence microscope (BZ-X700, Keyence) or a confocal microscope (SpinSR10, Olympus). All quantifications of immunofluorescence images were performed manually using ImageJ Software (Schneider et al., 2012).

“AChR cluster” is defined as group of α-BTX binding sites on the same MHC-positive SMC that are localized within an area of 1,000 μm^2^ (Wang et al., 2008). AChR clusters associated with nerve terminals are defined as “NMJs.” The “NMJ area” was defined as the area enclosed by the outermost α-BTX binding sites within an NMJ. The number and area of AChR clusters and NMJs were manually quantified using ImageJ software. The area of each NMJ was measured by selecting the area with the Polygon Selection Tool and then using the Analyze Particles command. The average area was calculated, and the distribution of each area was graphed as a histogram.

### RT-qPCR

Total RNAs were isolated using NucleoSpin® RNA Plus XS (740990, MACHEREY-NAGEL) according to the manufacturer’s protocol. The cDNA was synthesized using the QuantAccuracy®, RT-RamDA® cDNA Synthesis Kit (RMQ-101 T, TOYOBO). Quantitative PCR (qPCR) was performed using the Applied Biosystems QuantStudio 3 System and a standard SYBR Green detection protocol. Data were analyzed using the delta-Ct method and normalized to CSNK2A2 expression (Kawada et al., 2023). Primers used in this study are listed in Supplementary Table S6.

### Intracellular Ca^2+^ assay

The Calcium Kit-Fluo 4 (CS22, Dojindo Molecular Technologies) was used, following the manufacturer’s protocol, to measure intracellular Ca^2+^ (Gee et al., 2000). The cells were incubated in medium containing Fluo 4-AM for 1 h in a CO_2_ incubator to load the dye. Intracellular Ca^2+^ levels were monitored by time-lapse imaging under a fluorescence microscope. The sequence of images captured using time-lapse imaging was manually analyzed using ImageJ software. The ROI was set using the Polygon Selection Tool. The intensity of the cells was measured using the Measure command.

### Statistical analysis

All statistical analyses were performed in R software (R Core Team, 2025). Statistical results were presented as Mean +/− SD. After testing for normality using the Shapiro–Wilk normality test, Student’s t-test was used to analyze the number and area of AChR clusters and NMJs. In RT-qPCR, one-way analysis of variance followed by Tukey’s *post hoc* were used to analyze gene expression. If normality was rejected, a Wilcoxon rank-sum exact test was performed. *p*-value adjustment by Bonferroni method. *p* < 0.05 was considered statistically significant.

## Result

### Differentiation of MNs and SMCs from human iPSCs

The overall timeline of NMJ formation culture is shown in Figure 1B. To facilitate NMJ formation in cocultures of human iPSC-derived MNs and SMCs, we first optimized the differentiation conditions to obtain MNs and SMCs efficiently from human iPSCs. For MN differentiation, we used the human iPS cell line 454E2 maintained under feeder-free conditions and induced neurosphere formation. Eleven days after the initiation of differentiation, the neurospheres were dissociated and replated onto new culture dishes (Figure 2A). By day 32, we found that 62.3+/−7.8% (Mean +/− SD) and 69.5+/−11.6% of Neurofilament M (NFM)-positive cells were positive for ISLET1 (ISL) (Figures 2B,C) and HB9 (Figures 2D,E), respectively.

**Figure 2:**
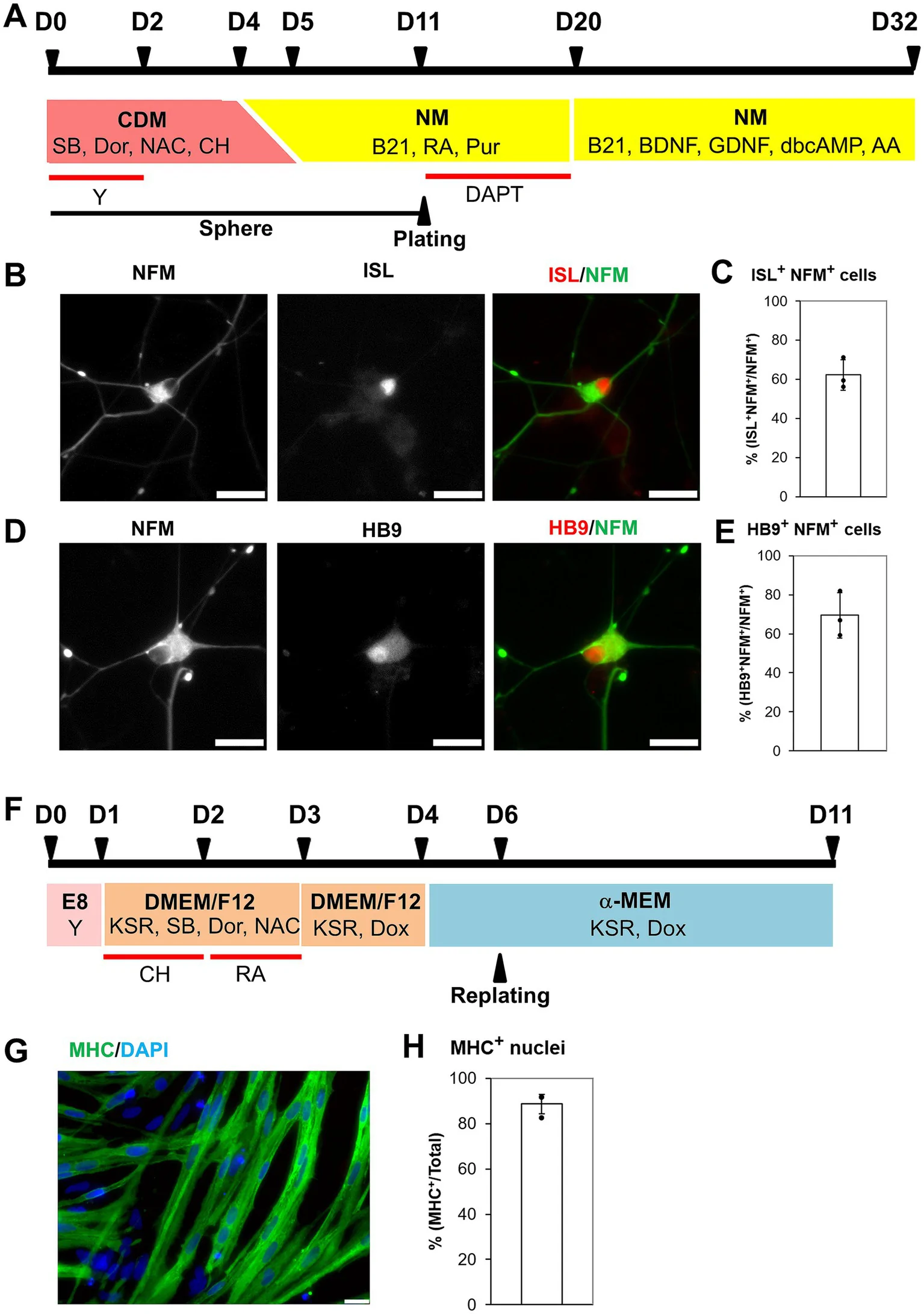
Differentiation of MNs and SMCs from human iPSCs. **(A)** Schematic representation of the time course for MN differentiation from human iPSCs. SB: SB431542 (a selective ALK inhibitor), Dor: Dorsomorphin (a selective ALK inhibitor), CH: CHIR99021 (a GSK-3 inhibitor), Y: Y27632 (a Rock inhibitor), Pur: Purmorphamin (a Smoothened receptor agonist), NAC: N-acetyl cysteine, B21: NeuroBrew-21, RA: retinoic acid, AA: ascorbic acid. **(B–E)** Differentiation of MNs from human iPSCs. **(B,D)** Representative fluorescence image of differentiated MNs. The differentiated MNs were immunostained for NFM together with ISL **(B)** or HB9 (D) on day 32. Scale bar: 25 μm. **(C)** The ratio of the number of ISL /NFM double positive cells to that of NFM-positive cells. **(E)** The ratio of the number of HB9 /NFM double positive cells to that of NFM-positive cells. Mean +/− SD (*n* = 3) are indicated. **(F)** Schematic representation of the time course for SMC differentiation from Dox-inducible MYOD1-expressing human iPSCs. **(G)** A representative fluorescence image of differentiated SMCs. The differentiated SMCs were immunostained for MHC, with nuclear counterstaining by DAPI, at day 11. Scale bar: 25 μm. **(H)** The ratio of the number of MHC-positive nuclei to that of all nuclei. Mean +/− SD (*n* = 3) are indicated.

For SMC differentiation, we employed a Dox-inducible MYOD1 expression vector, as this method enables rapid and highly efficient differentiation into SMCs (Uchimura et al., 2017). Three days after the cloned MYOD1-inducible iPSCs were seeded, Dox was added to the culture medium to induce MYOD1 expression. The cells were then replated onto new culture dishes on day 6 (Figure 2F). We found that 88.7+/−4.3% of nuclei were surrounded by cytoplasm immunostained for myosin heavy chain (MHC), a marker of SMCs (Figures 2G-H).

### NMJ formation by compartmentalized culture

To demonstrate the quantitative reproducibility of NMJ formation in cocultures of human iPSC-derived MNs and SMCs, it was necessary to generate countable numbers of morphologically identifiable NMJs within a restricted culture area. Therefore, we aimed to achieve a high frequency of NMJ formation. To accomplish this, we hypothesized that establishing coculture conditions that closely mimic physiological NMJ development would be important. To reproduce the developmental context, we employed a compartmentalized culture system (Campenot chamber), in which the cell bodies of MNs and SMCs are separated until MNs extend their axons along scratched surfaces of the culture dish through separating slits and subsequently contact SMCs (Figures 3A,B) (Campenot, 1977). Neurospheres (day 11 of the MN differentiation procedure shown in Figure 2A) were dissociated, seeded into the slit of the chamber, and maintained in MN differentiation medium (MNM) (see Supplementary Tables S1, S2). Six days later, skeletal muscle cells (day 6 of the SMC differentiation procedure shown in Figure 2F) were transferred into the chamber compartment (Figures 3A,B; Supplementary Figure S1A). Thereafter, SMCs were cultured in skeletal muscle differentiation medium (SMM) (see Supplementary Tables S1, S3) in the chamber compartment, while MNs were maintained in MNM in the slit for 21 days (see Supplementary Table S4). Using this procedure, NMJ-like structures were identified by the presence of *α*-bungarotoxin (α-BTX) binding sites associated with neuritic structures which were detected by co-localization with neurofilament M (NFM) and synaptophysin (SF) immunostaining using confocal microscopy (Figure 3C). Because MN differentiation from SMC culture was previously reported (Lin et al., 2019), we examined compartmentalized cultures of SMCs without MNs to confirm that the NMJ-like structures are formed between the neurites extended from the “slit” and SMCs in the “compartment.” In these cultures, scattered α-BTX binding sites were detected on SMCs; however, no structures immunostained for NFM or SF were observed. These results indicate that axons extending from compartmentalized MNs form an NMJ-like axon-skeletal muscle contact site.

**Figure 3:**
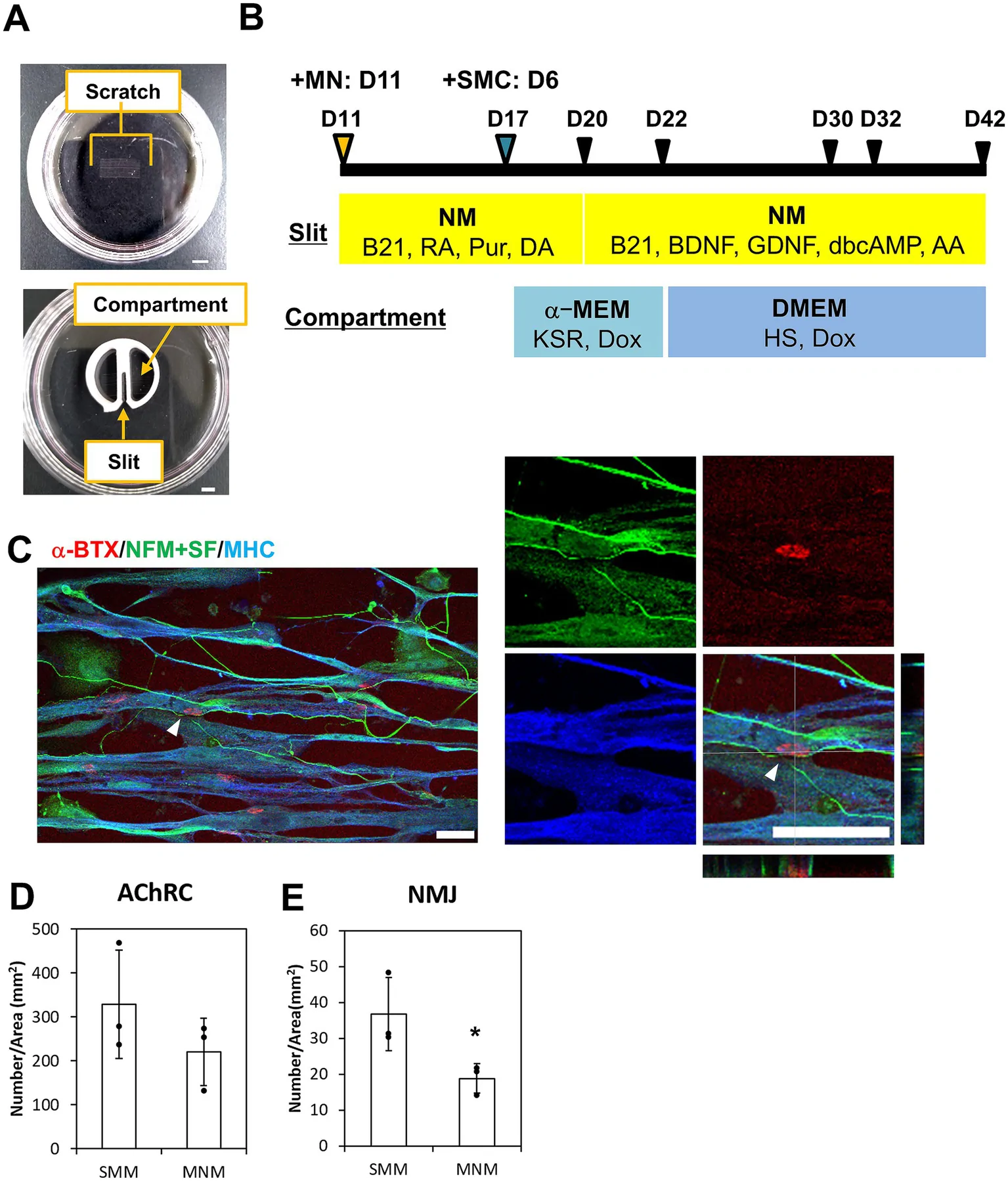
NMJ formation in a compartmentalized culture system. **(A)** Top: The scratch channels on Matrigel-coated 60 mm dishes. Bottom: The Campenot chamber, with “slit” and “compartments,” attached over the scratch channels. Scale bar: 5 mm. **(B)** Schematic representation of the time course for the NMJ formation culture using a Campenot chamber. The dispersed neurospheres were seeded into the “slit” of the chamber. Six days later, SMCs were seeded into the “compartments” of the chamber. Scale bar: 5 mm. DA: DAPT (a *γ*-secretase inhibitor). **(C)** Representative confocal image of NMJs formed in the culture. The cells in the “compartments” of the chamber were immunostained for NFM, SF, and MHC, and AChRs were visualized with a TMR-conjugated *α*-BTX, at day 32. The right panels show enlarged views of the left panel, with the *Z*-axis view sectioning the NMJs indicated by the arrowhead along the white lines. Scale bar: 20 μm. **(D,E)** The numbers of AChR clusters (AChRC) **(D)** and NMJs **(E)** per square millimeter of MHC-positive SMCs area under indicated conditions at D32. Three independent experiments were performed. Mean +/− SD (*n* = 3) are indicated. *p < 0.05 by Student’s *t*-test.

To demonstrate the quantitative reproducibility of NMJ formation, we repeated the same NMJ formation experiment three times using the same human iPSC clone as the starting material and counted *α*-BTX binding sites at synaptic terminals on MHC-positive SMCs within the scratched channels of the compartments. We found an average of 37 α-BTX binding sites at synaptic terminals per square millimeter of MHC-positive SMCs. Furthermore, the coefficient of variation was 0.27, indicating low variability (Supplementary Table S5). The total area of MHC-positive SMCs did not vary under culture conditions, and the coefficient of variation was 0.052 (Supplementary Figure S1B and Supplementary Table S5). The results of these three independent experiments suggest that our method for NMJ formation enables quantitatively reproducible measurements.

To verify the importance of the compartmentalized culture system in facilitating NMJ formation, we compared NMJ formation under two conditions: (1) MNs and SMCs cultured in their respective optimal media, and (2) both MNs and SMCs cultured in MNM alone. We found that, while there was no significant difference in the number of α-BTX binding sites, the number of NMJ-like structures was significantly reduced when MNM was used for both compartments and slit (Figures 3D,E). These results suggest that maintaining MNs and SMCs under their respective optimal culture conditions using a compartmentalized culture system is important for enhancing NMJ formation.

### Maturation of NMJ and myofiber

Maturation of NMJs during development in rodents is characterized by increased morphological complexity, with structures changing from relatively simple round or oval shapes to convoluted, so-called “pretzel-like” structures (Sanes and Lichtman, 1999). In humans, however, NMJs have been reported to exhibit a patchy morphology and to increase in the area of AChR clusters with increasing age (Oda, 1984; Jones et al., 2017). To examine whether NMJs formed in our culture system exhibit changes compatible with physiologically observed maturation, we maintained the NMJ cultures for up to day 42 and analyzed changes in NMJ number and morphology over time. We found that the number of *α*-BTX binding sites and NMJ-like structure gradually decreased during the maturation phase of the culture (Figures 4A,B). Morphological analysis of individual NMJ-like structures showed that the area of individual NMJs tends to increase as the culture period becomes longer (Figures 4C,D). These results suggest that morphological changes of NMJs observed in our culture system are compatible with the previously reported human NMJ maturation (Oda, 1984; Jones et al., 2017).

**Figure 4:**
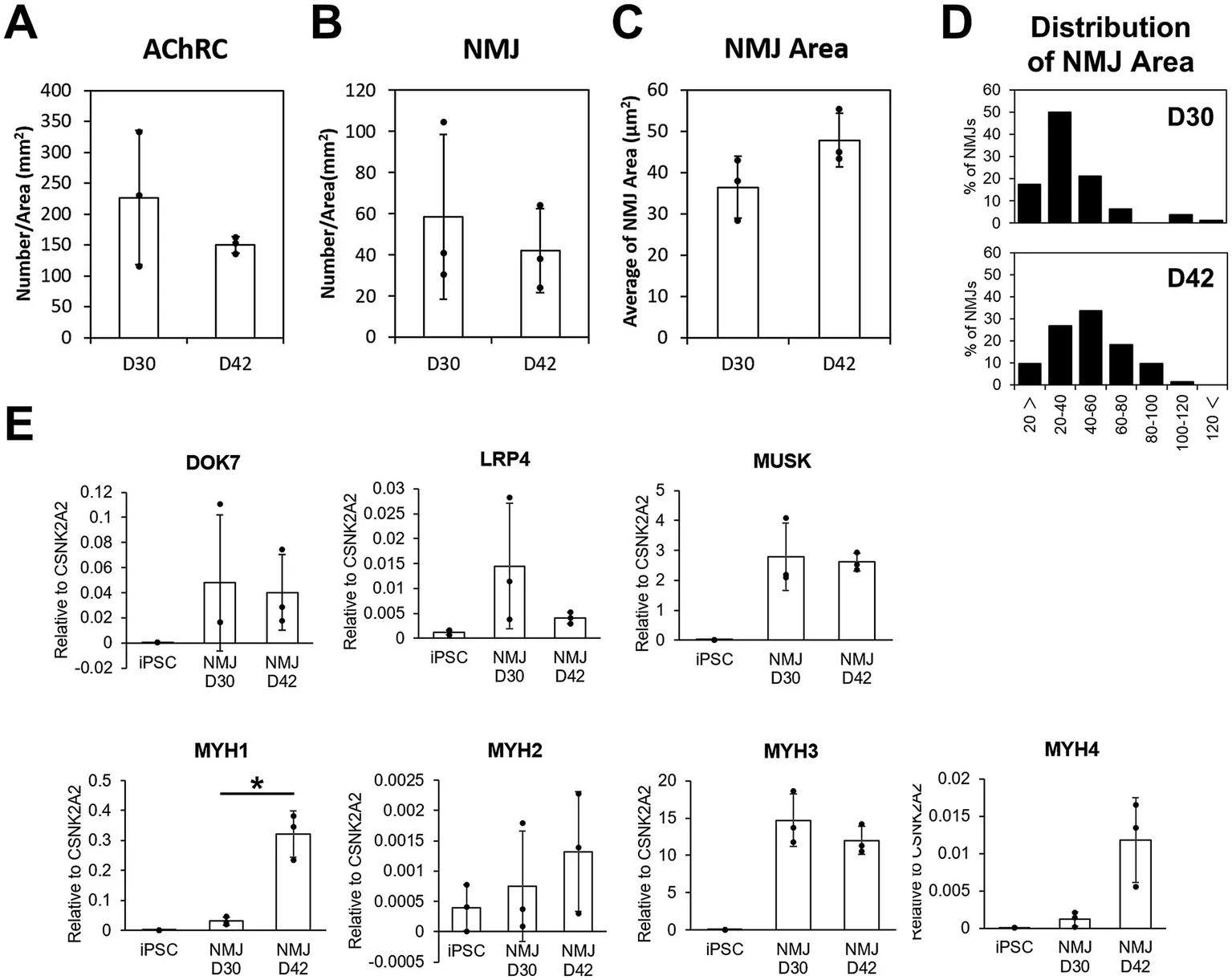
Maturation of the NMJ culture. **(A,B)** The bar graphs showing the number of AChR clusters (AChRC) **(A)** and NMJs **(B)** per unit MHC-positive cell area at indicated time points. Three independent experiments were performed. Mean +/− SD (*n* = 3) are indicated. No differences between D30 and D42 by Student’s t-test. **(C)** The bar graph showing the mean NMJ areas at indicated time points. Three independent experiments were performed. Mean +/− SD (*n* = 3) are indicated. No differences between D30 and D42 by Student’s *t*-test. **(D)** The histograms showing the distribution of the area of each NMJ at indicated time points. The area of NMJs is grouped in bins spanning 20 μm^2^. **(E)** NMJ and SMC-related gene expression at indicated time points quantified by RT-qPCR. Expression level of each gene is normalized to CSNK2A2 is shown. Three independent experiments were performed. Mean +/− SD (*n* = 3) are indicated. *p < 0.05 by Tukey or Wilcoxon test.

To correlate expression profile of genes responsible for NMJ formation and myotube maturation with the observed morphological changes of NMJs, we examined the expression of genes related to NMJ and myotube differentiation during extended culture. The genes include DOK7, LRP4, and MUSK, encoding key molecules involved in NMJ formation (Kummer et al., 2006; Kim et al., 2008) and signaling in myotubes, AChR subunits (Mishina et al., 1986), and myosin heavy chain gene family MYH3, representing the embryonic isoform, as well as MYH1, MYH2, and MYH4 which are more mature isoforms (Schiaffino and Reggiani, 2011). We found that the expression of these NMJ-related genes on the skeletal muscle side was elevated at day 30, which is consistent with the observation of α-BTX-positive clusters on day 30 as well (Figure 4E). We also detected induction of expression of the AChR components, including α, *γ*, and *ε* subunits, during the maturation of the culture (Supplementary Figure S2). With regard to the MYH family members, we found that expression of MYH3 gradually decreased, while MYH1 and MYH4 expression significantly increased during the maturation of the culture (Figure 4E). Collectively, these results suggest that NMJs formed in our culture system exhibit morphological and molecular characteristics consistent with physiological NMJ maturation (Mishina et al., 1986; Kummer et al., 2006; Kim et al., 2008; Schiaffino and Reggiani, 2011).

### Functional analysis of NMJ

To demonstrate that the morphologically identified NMJs formed in cocultures of human iPSC-derived MNs and SMCs are physiologically active, we examined changes in intracellular Ca^2+^ levels in SMCs in response to MN stimulation. For this purpose, Fluo-4AM., a fluorescent Ca^2+^ indicator, was loaded into SMCs, and MNs were stimulated by adding KCl to the “slit” of the chamber. Changes in Fluo-4AM. fluorescence in response to MN stimulation were monitored by time-lapse imaging (Figure 5A) (Gee et al., 2000). We found that some muscle cells exhibited transient increases in fluorescence, whereas others showed no response (Figures 5B,C, Supplementary Figure S3, and Supplementary Movies S1–S6). Subsequently, KCl was applied directly to the skeletal muscle chamber to confirm the viability of the muscle cells, which resulted in increased fluorescence in all cells. As a control experiment, KCl was applied to the slit chamber in cultures lacking neuronal cells, and no change in Ca^2+^ levels was observed in the SMCs. These results suggest that the morphologically identifiable NMJs formed in our coculture system are capable of transmitting neuronal excitation to SMCs (Sanchez and Stefani, 1978; Somlyo et al., 1981).

**Figure 5:**
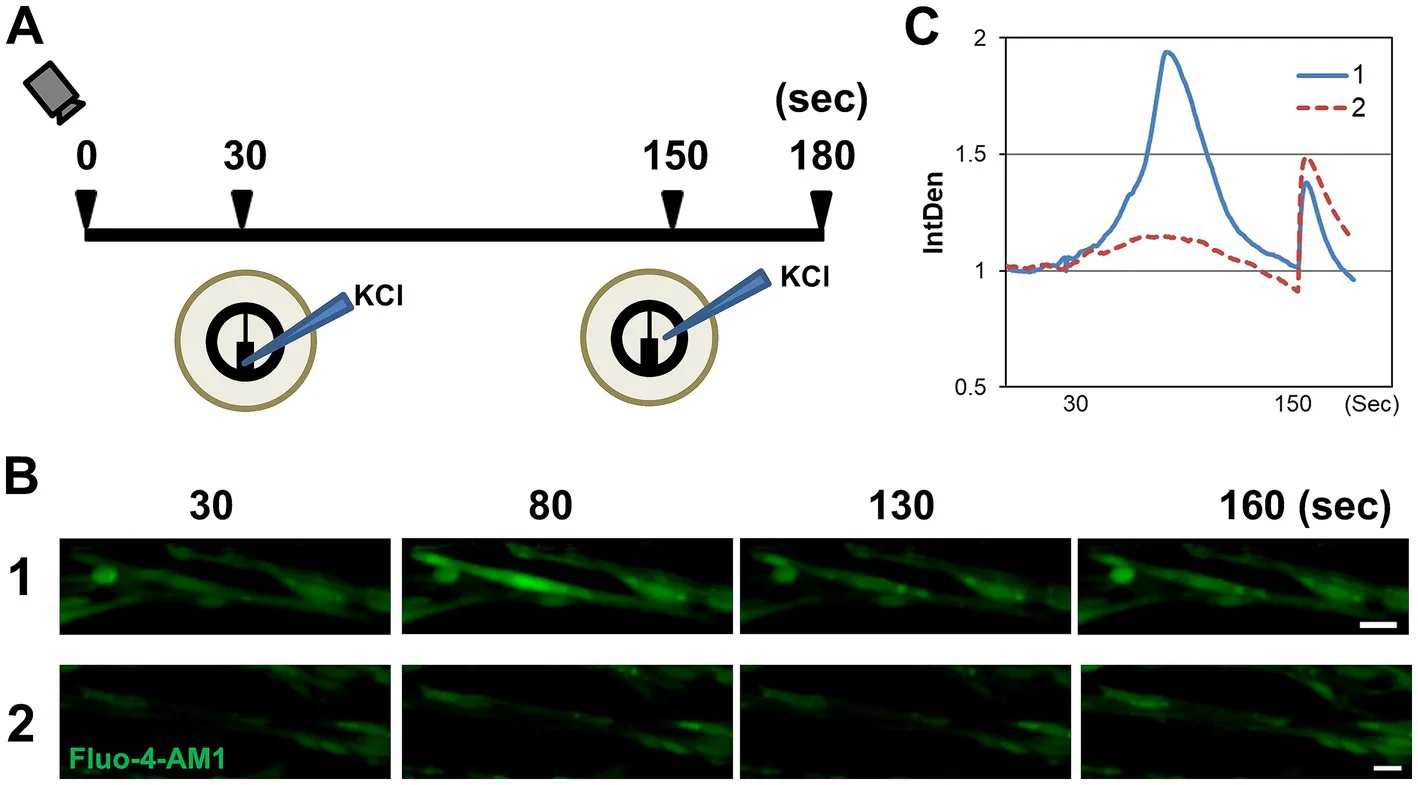
Myotubes in the NMJ formation culture show electrophysiological response to neural stimuli. **(A)** The time schedule of fluorescence time-lapse imaging of the Ca^2+^ response with Fluo4-AM dye. The cell bodies of MNs were stimulated by adding 50 mM KCl into the slit of the chamber at 30 s. The response of SMCs in the “compartment” was detected using fluorescence time-lapse imaging. The SMCs in the “chamber” were directly stimulated at 150 s to confirm viability of the myotubes. **(B)** Representative fluorescence images of SMCs at 30, 80, 130, and 160 s. The cells responsive (1) and unresponsive (2) to stimulation of MN cell bodies are shown. Fluo4-AM: A fluorescent indicator for Ca^2+^ ions. Scale bar: 20 μm. **(C)** Representative fluorescence intensity profile over time-lapse imaging. The fluorescence intensity relative to the intensity at time zero is shown. The blue line and red dotted line indicate the intensities of responsive and unresponsive cells, respectively.

## Discussion

An NMJ culture model using human-derived cells is valuable for disease modeling and drug development. In this study, we demonstrated quantitatively reproducible NMJ formation in culture using human iPSC-derived MNs and SMCs. NMJ formation in culture has long been studied using cells derived from various non-human model organisms (Fischbach, 1970; Peterson and Crain, 1970). An NMJ model using only human-derived cells was first reported in 2011 (Guo et al., 2011). In that study, MNs derived from spinal cord stem cells and primary skeletal myoblasts were used. Since 2015, several studies have reported NMJ models using human iPSC-derived MNs and muscle cells (Demestre et al., 2015; Yoshida et al., 2015). In these reports, morphologically identifiable NMJ structures and their electrophysiological properties were described. However, the quantitative reproducibility of NMJ formation was not addressed, and therefore applications such as testing drugs that affect the function of already formed synapses were mainly discussed. More recently, NMJ formation using organoid- and assembloid-based culture systems has also been reported (Andersen et al., 2020; Faustino Martins et al., 2020; Yin et al., 2025). Although these approaches more closely mimic physiological conditions, they have several limitations, including a lack of homogeneity, the absence of standardized protocols, and the complexity of three-dimensional imaging for morphological analysis of the generated culture (Yang et al., 2025). Organ-on-chip technology is another powerful tool for NMJ modeling and, like our method, employs compartmentalized devices (Uzel et al., 2016; Santhanam et al., 2018; Osaki et al., 2020; Fralish et al., 2021; Yamamoto et al., 2021). Their devices incorporate 3D culture and complex circuitry to closely resemble *in vivo* conditions, but their imaging techniques are also complex. Our simpler compartmentalized culture method may enable modeling of diseases that affect synaptogenic mechanisms, including CMS, as well as diseases that affect the maintenance of NMJs, including motor neuron diseases.

We employed a compartmentalized culture system to mimic the physiological developmental context in which extended axon tips contact myotubes. Previous studies have described several advantages of compartmentalized culture systems (Natarajan et al., 2019). Separation of neuronal and muscle cultures allows application of compounds or stimulation to only one side of the culture. Although mimicry of the physiological context has also been discussed, the difference between compartmentalized cultures and mixed cultures of MNs and SMCs in a single compartment has not been clearly demonstrated. In the present study, differentiation of MNs and SMCs under individually optimal conditions before extended axonal tips contact myotubes proved important for efficient NMJ formation. This observation may appear inconsistent with a previous report showing that application of GDNF to the muscle compartment increased NMJ formation (Zahavi et al., 2015). One possible explanation for this difference is the maturity of the muscle cells. The iPSC-derived muscle cells used in the present study are relatively immature and produce only low levels of neuronal survival factors such as GDNF, compared with fused myotubes used in the previous report, which produce higher levels of GDNF (Nguyen et al., 1998). When muscle cells produce sufficient levels of factors that support axonal survival, GDNF added to the muscle compartment medium may facilitate the extension of axon tips toward muscle cells (Zahavi et al., 2015). In our experiments, when extended motor axon tips survive in the presence of GDNF, they may fail to establish firm connections with muscle cells that produce only low levels of supportive factors including GDNF.

For skeletal muscle differentiation from human iPS cells, we used MYOD overexpression, a well-established approach for inducing skeletal muscle differentiation (Uchimura et al., 2017). Previous reports have shown that MYOD-overexpressing human iPS cells exhibit an immature skeletal muscle phenotype and tend to detach from the culture surface after 7–10 days, limiting long-term culture (Tanaka et al., 2013; Uchimura et al., 2017; Kawada et al., 2023). In contrast, in our NMJ coculture system, skeletal muscle cells in contact with MN neurites can be maintained for up to 2 months. Our observations suggest that MN-innervated skeletal muscle cells undergo maturation, likely reflecting the mutual dependence of MNs and muscle cells for survival and maturation under physiological conditions. Previous studies have shown that skeletal muscle maturation can be promoted by specific culture conditions or mechanical stimulation; here, we demonstrate that MN innervation itself can serve as a driving force for skeletal muscle maturation in culture (Nikolić et al., 2017; Maffioletti et al., 2018; Osaki et al., 2018). This point is important because differentiation of skeletal muscle cells from human stem cells through stepwise cytokine stimulation requires substantially longer periods compared with MYOD overexpression (Zhao et al., 2020). The enhanced maturation observed in our system likely contributes to the establishment of long-term coculture using a simplified culture method, and enables analysis of morphological maturation of NMJs.

A compartmentalized culture system was employed to mimic the physiological developmental context of MNs and SMCs. This approach appears successful at least in part, because increased numbers of NMJs were observed when this method was used, together with alterations in NMJ morphology and AChR subunit expression that may be consistent with physiological NMJ maturation (Oda, 1984; Mishina et al., 1986; Jones et al., 2017). Despite these observations, the progression of NMJ formation in our system differs from physiological NMJ development in several respects. First, Schwann cells were not included in our culture. Terminal or perisynaptic Schwann cells are known to play important roles in NMJ formation and maintenance (Sanes and Lichtman, 1999; Patrón and August, 2018). Although both our study and previous reports have shown that NMJs can form in the absence of Schwann cells, the NMJ formation process may not fully reflect physiological conditions without these cells (Guo et al., 2011; Marteyn et al., 2011; Demestre et al., 2015; Yoshida et al., 2015). Second, AChR clustering in our culture model appears different from that reported previously. In the *in vivo* cotext, axon terminals organize diffusely distributed AChRs into clusters at sites of nerve-muscle contact (Sanes and Lichtman, 1999; Cappello and Francolini, 2017). In culture models, however, spontaneous formation of AChR clusters independent of axonal contact is frequently observed (Wang et al., 2008; Cartaud et al., 2011). In *in vitro* NMJ formation models using human myoblasts as a source of SMCs, small spontaneously formed AChR clusters often merge into larger clusters in response to axonal contact, while many extrasynaptic clusters remain. In our NMJ formation model, spontaneous AChR clusters were less numerous than in models using human myoblasts, and AChR clusters tended to become larger at sites of axonal contact (Demestre et al., 2015; Bakooshli et al., 2019). It is possible to speculate that aneural AChR clusters may help guide infiltrating axons of MNs towards the correct location for NMJ development (Yang et al., 2001). However, the apparent differences between AChR clustering *in vivo* and in culture models using muscle cells differentiated from stem cells, including our model, suggest that the mechanisms underlying AChR cluster formation and maturation may differ. Future studies examining the detailed mechanisms of AChR clustering in our model, including the identification of molecules associated with AChRs during the progression of NMJ maturation, will be necessary to determine what types of diseases can be modeled using this NMJ formation system.

## Data Availability

The original contributions presented in the study are included in the article/Supplementary material, further inquiries can be directed to the corresponding author.
