# Supplementary material for: Neuromuscular junction formation by compartmentalized culture using human iPS cell-derived motor neurons and skeletal muscle cells
Source: Front Neural Circuits. 2026 Sep 17;20:1838276. doi: 10.3389/fncir.2026.1838276 (PMC13627353; doi:10.3389/fncir.2026.1838276)
Supplement: Supplementary file 1 [file Data_Sheet_1.PDF]

Supplementary Materials

1. List of Supplementary Data

Supplementary Figure S1. An overview of the compartmentalized culture (Related Figure 3A and 3C) and total area of MHC-positive cells (Related Figure 3D-E)

Supplementary Figure S2. Quantification of NMJ-related gene expression analyzed by RT-qPCR (Related Figure 4)

Supplementary Figure S3. Fluorescence time-lapse imaging of the Ca2+ response with Fluo4-AM dye (Related Figure 5)

Supplementary Table S1. Composition of medium (Related Figures 1, 2, and 3)

Supplementary Table S2. MN differentiation medium (MNM) (Related Figures 1, 2, and 3)

Supplementary Table S3. SMC differentiation medium (SMM) (Related Figures 1, 2, and 3)

Supplementary Table S4. MNM and SMM in NMJ formation (Related Figures 1, 2, and 3)

Supplementary Table S5. Statistical values of NMJ formation (Related Figure 3)

Supplementary Table S6. Primer list (Related Figure 4 and Supplementary Figure S1)

Supplementary Movie S1. Fluorescence time-lapse imaging of the Ca2+ response with Fluo4-AM dye (Related Figure 5C-1)

Supplementary Movie S2. Fluorescence time-lapse imaging of the Ca2+ response with Fluo4-AM dye (Related Figure 5C-2)

Supplementary Movie S3. Fluorescence time-lapse imaging of the Ca2+ response with Fluo4-AM dye (Related Supplementary figure S3A-3)

Supplementary Movie S4. Fluorescence time-lapse imaging of the Ca2+ response with Fluo4-AM dye (Related Supplementary figure S3A-4)

Supplementary Movie S5. Fluorescence time-lapse imaging of the Ca2+ response with Fluo4-AM dye (Related Supplementary figure S3B-5)

Supplementary Movie S6. Fluorescence time-lapse imaging of the Ca2+ response with Fluo4-AM dye (Related Supplementary figure S3B-6)

2. Supplementary Figures

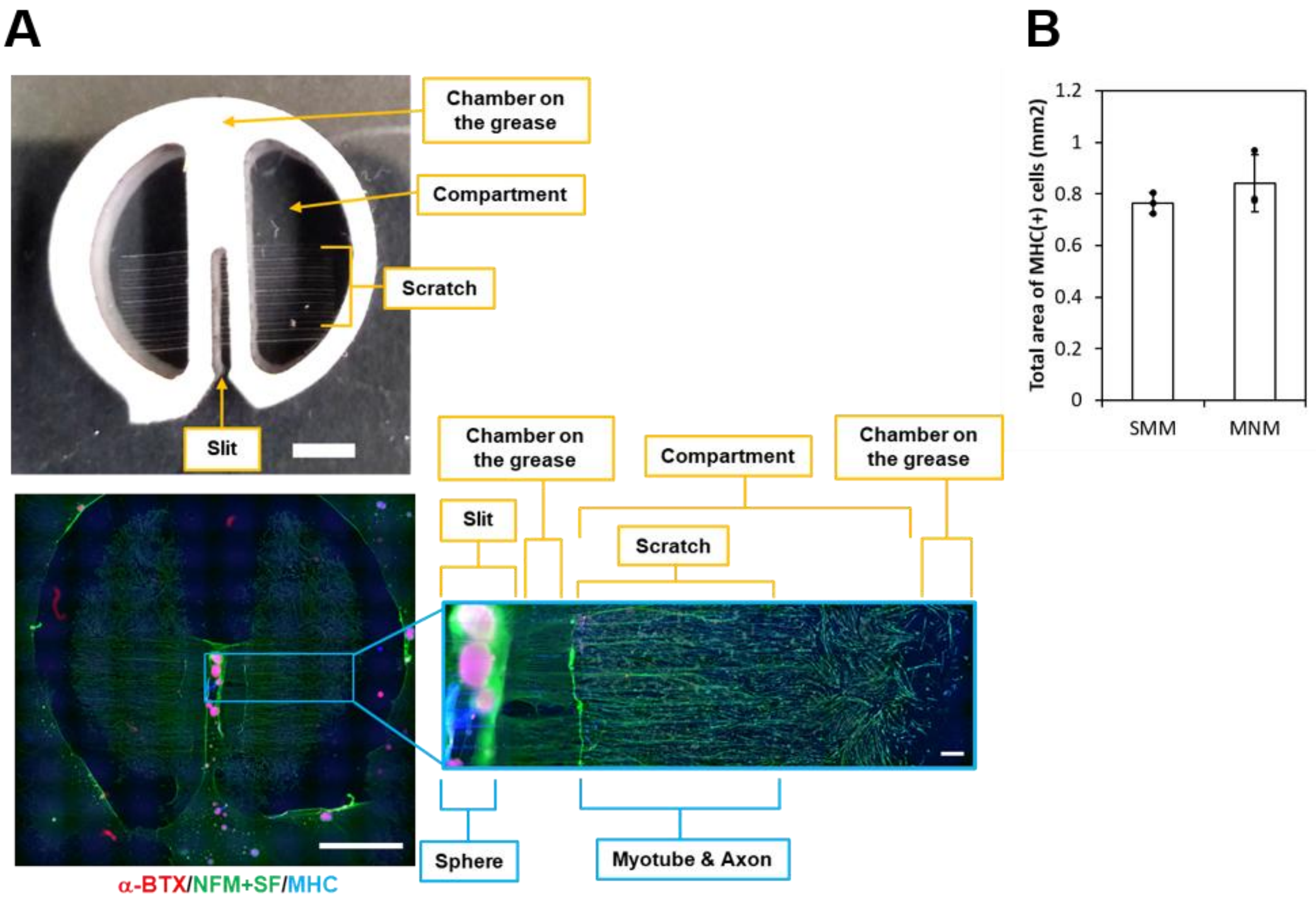

Supplementary Figure S1. (A) An overview of the compartmentalized culture. Top: the Campenot chamber, with "slit" and "compartments", attached over the scratch channels. Scale bar: 5 mm. (Enlarged view of Figure 3A) Bottom: A representative fluorescence image of NMJs formed in the culture. The cells of the chamber were immunostained for NFM, SF, and MHC, and AChRs were visualized with a TMR-conjugated α-BTX, at day 36. The right panels show enlarged views of the left panel. Scale bar: 400 µm. (B) Total area of MHC-positive cells. The total area of MHC-positive cells under the indicated conditions. Three independent experiments were performed. Mean +/- SD (n=3) are indicated. No differences between SMM and MNM by Student's t-test.

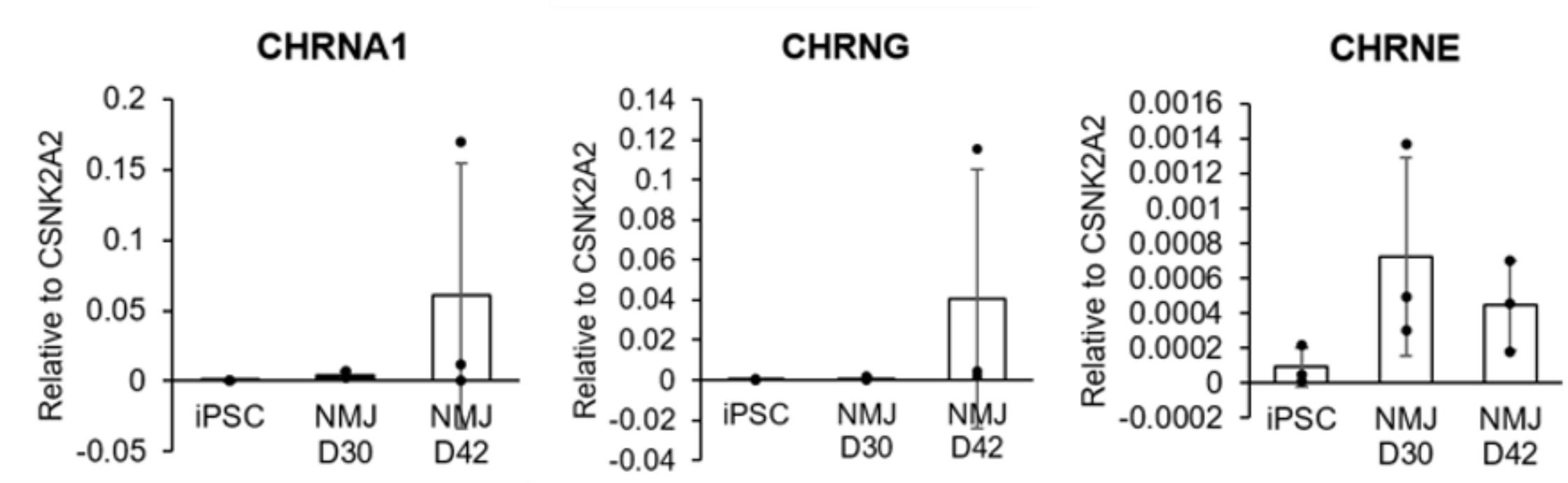

Supplementary Figure S2. Quantification of NMJ-related gene expression analyzed by RT-qPCR. Expression levels of each gene normalized to CSNK2A2 are shown. Three independent experiments were performed. Mean +/- SD (n=3) are indicated. No differences between D30 and D42 by Tukey's test or Wilcoxon test.

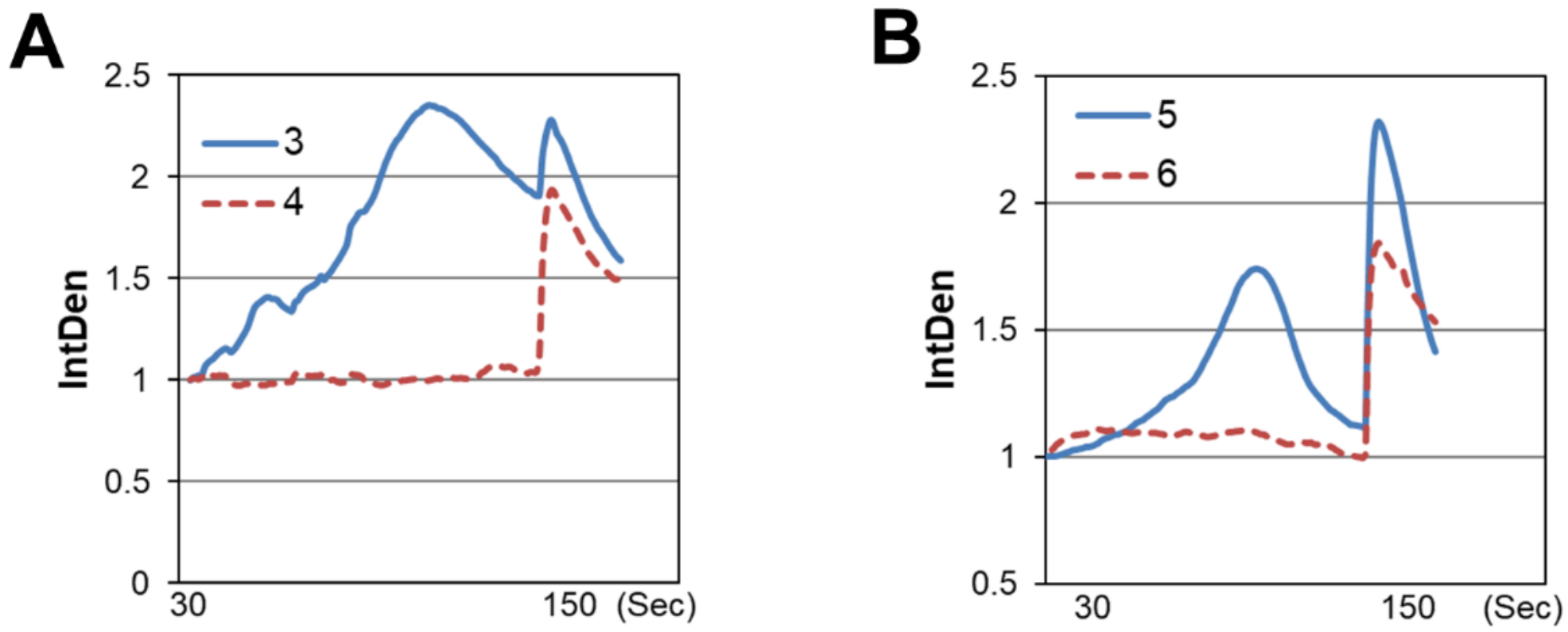

Supplementary Figure S3. Fluorescence time-lapse imaging of the Ca^{2+} response with Fluo4-AM dye. (A, B) Representative fluorescence intensity profiles over time-lapse imaging. The fluorescence intensity relative to the intensity at time zero is shown. The blue line and red dotted lines indicate the intensities of responsive and unresponsive cells, respectively. (A), (B), and Figure 5C were independent experiments.

3. Supplementary Tables

Supplementary Table S1. Composition of medium

Component(s)	Abbreviation	Catalogue	Source
Essential 8 Flex medium	E8	A28585	Thermo Scientific
Chemical defined medium	CDM	-	KOHJIN BIO
Neuro medium	NM	130-093-570	Miltenyi Biotec
DMEM/F12		11330032	Thermo Scientific
α-MEM		21445-95	Nacalai tesque
DMEM		044-29765	WAKO
SB431542	SB	04-0010	STEMGENT
Dorsomorphin	Dor	3093	TOCRIS
N-acetyl cysteine (NAC)	NAC	A7250	SIGMA
CHIR99021	CH	4423	TOCRIS
Y27632	Y	Y-5301	LC Laboratories
Retinoic acid	RA	R2625	SIGMA
Purmorphamin	Pur	10009634	Cayman Chemical
NeuroBrew-21	B21	130-093-566	Miltenyi Biotec
DAPT		3219-v	PEPTIDE INSTITUTE
BDNF		48-BDB	R&D systems
GDNF		212-GD	R&D systems
dbcAMP		11540-74	Nacalai tesque
Ascorbic acid (2-Phospho-L-ascorbic acid trisodium salt)	AA	49752	SIGMA
Knockout Serum Replacement	KSR	10828028	Thermo Scientific
Doxycycline	Dox	D5897	LKT labs
Horse Serum	HS	16050-122	Life Technologies

Supplementary Table S2. MN differentiation medium (MNM)

Day	D0-D1	D2-D3	D4	D5-D11	D12-D19	D20~
Medium	MNM1	MNM2	MNM3	MNM4	MNM5	MNM6
Basal medium	CDM	CDM	CDM: NM (1:1)	NM	NM	NM
Components	10 μM SB	10 mM SB	0.1 μM RA	1x B21	1x B21	1x B21
	2 μM Dor	2 mM Dor	1 μM Pur	0.1 μM RA	0.1 μM RA	10 ng/mL BDNF
	0.5 mM NAC	0.5 mM NAC		1 μM Pur	1 μM Pur	10 ng/mL GDNF
	3 μM CH	3 mM CH			10 μM DAPT	500 μM dbcAMP
	10 μM Y					200 μM AA

Supplementary Table S3. SMC differentiation medium (SMM)

Day	D0	D1	D2	D3	D4-D11
Medium	E8	SMM1	SMM2	SMM3	SMM4
Basal medium	E8	DMEM/F12	DMEM/F12	DMEM/F12	α-MEM
Components	10 μM Y	5% KSR	5% KSR	5% KSR	5% KSR
		10 μM SB	10 μM SB	1 μg/mL Dox	1 μg/mL Dox
		2 μM Dor	2 μM Dor		
		0.5 μM NAC	0.5 mM NAC		
		3 μM CH	0.1 μM RA		

Supplementary Table S4. MNM and SMM in NMJ formation

Outside of compartment (Slit side)		
Day	D11-D19	D20-D42
Medium	MNM5	MNM6
Basal medium	NM	NM
Components	1x B21	1x B21
	0.1 μM RA	10 ng/mL BDNF
	1 μM Pur	10 ng/mL GDNF
	10 μM DAPT	500 μM dbcAMP
		200 μM AA
Inside of compartment		
Day	D17-D21	D22-D42
Medium	SMM4	SMM5
Basal medium	α-MEM	DMEM
Components	5% KSR	5% HS
	1 μg/mL Dox	1 μg/mL Dox

Supplementary Table S5. Statistical values of NMJ formation

	#AChRC/Area		#NMJ/Area		Total Area of MHC(+) cell (mm^2)	
	SMM	MNM	SMM	MNM	SMM	MNM
**Mean**	328.1456	219.7606	36.75405	18.82992	0.764300554	0.840331753
**Variance**	15253.12	5885.383	103.3612	16.88621	0.012340749	0.001629285
**Standard Deviation**	123.5035	76.71625	10.16667	4.109284	0.040364401	0.111088924
**Coefficient of Variation (CV)**	0.376368	0.34909	0.276614	0.218232	0.05281221	0.132196509

Supplementary Table S6. Primer list

Primers	Sequence (5′-3′)
Human CSNK2A2	AGGAGCAGGGAGAGACTGAG
	AATGTAGCACCTCAGTGGGC
Human DOK7	TGCCAAGCGGATTCATCTTTG
	GACGATGCAGTCGAACAGGAA
Human LRP4	GTGCTCTTGGAGAGTGTACCTG
	AAGAGGGGAACAGGTAGGTAG
Human MUSK	CTGGTTGCCTTCAGCGGAA
	CTGCACACATGAAAGTAGCCA
Human MYH1	CTGGTGGACAAACTGCAAGC
	TGGATCCTGCGGAATTTGGAG
Human MYH2	CTGAGGGAGGAGCGACTCT
	CTCGGGCTTATACACAGGCA
Human MYH3	GCAGATTGAGCTGGAAAAGG
	TCAGCTGCTCGATCTCTTCA
Human MYH4	TGCAATGAAGACTCTGGCTTTC
	TTTTTGCCACCTTTCTTTCCA
Human CHRNA1	GCCCAGACCTTGTTCTCTATAAC
	GGGTGACGATGATCTCACAGTA
Human CHRNG	CTGGCAGAACTGCTCCCTTA
	CAGGAGCATCTTGGCTGGTC
Human CHRNE	CCAGACCGTCTTCTTGTTCCT
	TTGAGCACGATGACGCAATTC
